# Genetic Diversity of the *Mycobacterium tuberculosis* Beijing Family Based on SNP and VNTR Typing Profiles in Asian Countries

**DOI:** 10.1371/journal.pone.0039792

**Published:** 2012-07-12

**Authors:** Yih-Yuan Chen, Jia-Ru Chang, Wei-Feng Huang, Shu-Chen Kuo, Ih-Jen Su, Jun-Ren Sun, Tzong-Shi Chiueh, Tsi-Shu Huang, Yao-Shen Chen, Horng-Yunn Dou

**Affiliations:** 1 National Institute of Infectious Diseases and Vaccinology, National Health Research Institutes, Zhunan, Miaoli, Taiwan; 2 Division of Clinical Pathology, Department of Pathology, Tri-Service General Hospital and National Defense Medical Center, Taipei, Taiwan; 3 Department of Microbiology, Kaohsiung Veterans General Hospital, Kaohsiung, Taiwan; 4 School of Medical Laboratory Science and Biotechnology, Taipei Medical University, Taipei, Taiwan; University of Delhi, India

## Abstract

The *Mycobacterium tuberculosis* (MTB) Beijing strain is highly virulent, drug resistant, and endemic over Asia. To explore the genetic diversity of this family in several different regions of eastern Asia, 338 Beijing strains collected in Taiwan (Republic of China) were analyzed by mycobacterial interspersed repetitive unit-variable number tandem repeat (MIRU-VNTR) typing and compared with published MIRU-VNTR profiles and by the Hunter-Gaston diversity index (HGDI) of Beijing strains from Japan and South Korea. The results revealed that VNTR2163b (HGDI>0.6) and five other loci (VNTR424, VNTR4052, VNTR1955, VNTR4156 and VNTR 2996; HGDI>0.3) could be used to discriminate the Beijing strains in a given geographic region. Analysis based on the number of VNTR repeats showed three VNTRs (VNTR424, 3192, and 1955) to be phylogenetically informative loci. In addition, to determine the geographic variation of sequence types in MTB populations, we also compared sequence type (ST) data of our strains with published ST profiles of Beijing strains from Japan and Thailand. ST10, ST22, and ST19 were found to be prevalent in Taiwan (82%) and Thailand (92%). Furthermore, classification of Beijing sublineages as ancient or modern in Taiwan was found to depend on the repeat number of VNTR424. Finally, phylogenetic relationships of MTB isolates in Taiwan, South Korea, and Japan were revealed by a minimum spanning tree based on MIRU-VNTR genotyping. In this topology, the MIRU-VNTR genotypes of the respective clusters were tightly correlated to other genotypic characters. These results are consistent with the hypothesis that clonal evolution of these MTB lineages has occurred.

## Introduction

Tuberculosis (TB) remains a major infectious and deadly disease in many parts of the world. It is an immense public health problem in Taiwan (Republic of China) despite a steady decrease in both incidence and mortality rates since 1950. Among the most prevalent *Mycobacterium tuberculosis* (MTB) strains worldwide is the Beijing genotype, which has spread from its origin in Asia to countries of the former Soviet Union, Europe, Africa and the United States, often in association with hypervirulence and multiple drug resistance [1], [2], [3], [4], [5]. The Beijing genotype was first described from isolates in the Beijing region and Mongolia in 1995 [6] and is defined by its highly conserved spoligotype patterns and characteristic IS*6110* RFLP profiles.

Beijing strains can be further divided into ancestral, modern and W subfamilies based on the presence or absence of transposon IS*6110* insertions in the NTF chromosomal region [7], [8], [9]. The modern subfamily contains a single IS*6110* insertion upstream of the NTF region. It is reportedly dominant worldwide, leading to speculation that the modern subfamily has higher virulence and transmissibility than the other two subfamilies [4], [10]. The W subfamily possesses two IS*6110* insertions in the NTF region. In contrast to the worldwide prevalence of the modern subfamily, W strains are dominant in the United States, where they have been responsible for outbreaks of multidrug resistant TB [3], [11]. The ancestral subfamily is identified by the absence of IS*6110* insertions within the NTF region. Compared with W strains, this subfamily is highly diverse and has caused small-scale outbreaks in the United States [12]. In the last dozen years, discrimination of MTB strains has been achieved by the use of PCR-based methods, including spoligotyping and MIRU-VNTR typing [11], [13].

Our previous studies reported that the Beijing genotypes in the Taipei region of Taiwan mainly belong to the modern subfamily [14], [15], [16]. In contrast, ancient Beijing strains are dominant in Japan [17], [18]. However, it is not known whether the observed variability (modern versus ancient) is due to a selective advantage of particular Beijing strains over other MTB genotypes or to geographical differences among human populations. Here we analyzed a total of 338 Beijing strains collected from four geographical regions in Taiwan by MIRU-VNTR typing and synonymous single nucleotide polymorphism (SNP) typing. We then compared our results against published profiles from South Korea, Japan and Thailand to estimate potential epidemiological linkage in the region.

## Results

### Discriminatory Power of VNTR Markers in Taiwan

A total of 338 Beijing strains was collected from 5 general hospitals located in four geographical regions of Taiwan, including northern (Taipei Tri-Service General Hospital), eastern (Mennonite Christian Hospital), central (Wan-Chiao Hospital) and southern regions. The copy number of each MIRU-VNTR locus was found to vary from 1 to over 15 (Table 1), and the HGDI values representing the discriminatory power of each locus are shown in Table 2. Among the 24 loci, the discriminatory index for two loci (VNTR424, VNTR2163b) was high (HGDI >0.6), according to the definition of Sola et al. [19]. Seven loci (VNTR154, VNTF3192, VNTR4052, VNTR1955, VNTR4156, VNTR 2996 and VNTR960) were dispersed and had moderate discriminatory power (0.3≤ HGDI ≤0.6). Fifteen loci (VNTR2347, VNTR2059, VNTR2687, VNTR2531, VNTR3007, VNTR580, VNTR2461, VNTR1644, VNTR577, VNTR2401, VNTR3171, VNTR4348, VNTR3690, VNTR2165 andVNTR802) had poor discriminatory power (HGDI<0.3).

**Table 1:** Frequency of MIRU-VNTR alleles and allelic diversity at each locus in *M. tuberculosis* isolates from Taiwan.

MIRU-VNTRlocus	No. of isolates with indicated MIRU allele^a^																No. AV^b^
	0	1	2	3	4	5	6	7	8	9	10	11	12	13	14	>15	
VNTR2347 (Mtub29)		1	1	336													3
VNTR2059 (MIRU20)		2	336														2
VNTR2687 (MIRU24)		335	3														2
VNTR2531 (MIRU23)		1				335	2										3
VNTR3007 (MIRU27)		1	1	334	2												4
VNTR580 (MIRU04)	3	2	330	1	1	1											6
MIRU2461 (ETR-B)		5	328	4			1										4
VNTR1644 (MIRU16)			8	323	7												3
VNTR577 (ETR-C)			3	3	321	11											4
VNTR2401 (Mtub30)		18	319	1													3
VNTR3171 (Mtub34)			11	312	13	2											4
VNTR4348 (MIRU39)		3	13	303	19												4
VNTR3690 (Mtub39)		2	2	300	24	9		1									6
VNTR2165 (ETR-A)			1	39	296	1										1	5
VNTR802 (MIRU40)		14	17	297	9	1											5
VNTR154 (MIRU02)			156	2	10	164	5		1								6
VNTR960 (MIRU10)		6	51	273	5	2		1									6
VNTR2996 (MIRU26)		2	1	1	9	10	49	244	14	3	4	1					11
VNTR4156 (QUB4156)		4	58	209	61	5											5
VNTR1955 (Mtub21)		1	3	12	126	184	3	3	4	1	1						10
VNTR4052 (QUB26)		2	3	7	4	20	38	216	33	6	5	4					11
VNTR3192 (MIRU31)		1	11	175	17	128	4	2									7
VNTR424 (Mtub04)		5	6	50	125	148	4										6
VNTR2163b (QUB11b)		1	4	22	31	100	145	26	3	1	2					2	11

a N = 338.

b AV, allelic variants.

**Table 2:** HGDI values of *M. tuberculosis* isolates from Taiwan, Japan and South Korea.

MIRU-VNTRlocus	Taiwan	Japan [18]	SouthKorea [20]
VNTR2347 (Mtub29)	0.011	N.D.	0.118
VNTR2059 (MIRU20)	0.012	0.05	0.108
VNTR2687 (MIRU24)	0.017	0	0
VNTR2531 (MIRU23)	0.017	0.146	0.025
VNTR3007 (MIRU27)	0.024	0.163	0.182
VNTR580 (MIRU04)	0.047	0.05	0.049
MIRU2461 (ETR-B)	0.058	N.D.	0.025
VNTR1644 (MIRU16)	0.086	**0.307**	0.153
VNTR577 (ETR-C)	0.097	0.028	0.049
VNTR2401 (Mtub30)	0.107	**0.317**	0.152
VNTR3171 (Mtub34)	0.146	N.D.	0.0722
VNTR4348 (MIRU39)	0.192	0.183	0.277
VNTR3690 (Mtub39)	0.207	0.185	**0.463**
VNTR2165 (ETR-A)	0.22	0.145	0.12
VNTR802 (MIRU40)	0.224	0.269	**0.528**
VNTR960 (MIRU10)	**0.325**	**0.436**	0.178
VNTR2996 (MIRU26)	**0.456**	**0.341**	**0.521**
VNTR154 (MIRU02)	**0.552**	0	0.037
VNTR4156 (QUB4156)	**0.554**	**0.622**	**0.628**
VNTR1955 (Mtub21)	**0.565**	**0.459**	**0.614**
VNTR4052 (QUB26)	**0.566**	**0.675**	**0.707**
VNTR3192 (MIRU31)	**0.587**	0.296	**0.479**
VNTR424 (Mtub04)	**0.651**	**0.474**	**0.698**
VNTR2163b (QUB11b)	**0.71**	**0.767**	**0.737**

Numbers in bold indicate HGDI >0.3.

N.D.  =  not determined.

### Comparison of VNTR and ST profiles from Taiwan, South Korea, Japan and Thailand

To determine whether certain MIRU-VNTR loci could be used to discriminate Beijing strains, we analyzed published MIRU profiles and calculated the HGDI of Beijing strains from two other East Asian countries, Japan [18] and South Korea [20] (Table 2). Among the loci, the discriminatory index of VNTR2163b was greater than 0.6 in Taiwan, Japan and South Korea, whereas the HGDIs of five loci (VNTR 424, VNTR 4052, VNTR1955, VNTR 4156 and VNTR 2996) were higher than 0.3. Furthermore, the HGDIs of three loci (VNTR960, VNTR2401 and VNTR1644) from Japan and two loci (VNTR802 and VNTR 3690) from South Korea were also greater than 0.3.

To better understand the extent of geographic variation of the sequence types in MTB isolate populations, we also analyzed the ST profiles of Beijing strains from Taiwan (present study) (Table S1), Japan [18] and Thailand [21] (Table 3). Eight-hundred and fifty-six Beijing strains from these three studies were classified into 11 ST types, with 402 ancient and 454 modern Beijing isolates (Table 3). The proportion of ST10 was significantly higher in Taiwan (53%) and Thailand (58%) than in Japan (17%). As a group, the three major Beijing sublineages ST10, ST22, and ST19 were found to be prevalent in Taiwan (82%) and Thailand (92%). In contrast, ST3, STK, and ST19 were found to be prevalent in Japan (69%).

**Table 3:** Distribution features of sequence types of *M. tuberculosis* isolates from Taiwan, Japan and Thailand.

Beijingsublineage^a^	No. (%) strains		
	Taiwan	Japan [18]	Thailand [21]
ST11	4(1.18)	4(1.13)	
ST26	27(7.99)	28(7.89)	3(1.84)
ST3	13(3.85)	**84(23.66)**	
STK	4(1.18)	**51(14.37)**	3(1.84)
ST19	**50(14.79)**	**111(31.27)**	**28(17.18)**
ST25	7(2.07)	2(0.56)	
ST22	**49(14.50)**	14(3.94)	**28(17.18)**
ST10	**180(53.25)**	61(17.18)	**94(57.67)**
ST8			5(3.07)
STN	4(1.18, M)		
STF			2(1.23)
Ancient^b^	88(26.04)	280(78.87)	34(20.86)
Modern	250(73.96)	75(21.13)	129(79.14)
Total	338	355	163

a Sequence type of Beijing sublineages.

b The proportion of ancestral or modern Beijing strains was calculated by determining the presence of an IS*6110* insertion in the NTF chromosomal region.

### Evolution of Beijing Sublineages in Taiwan and Japan

**Figure 1:**
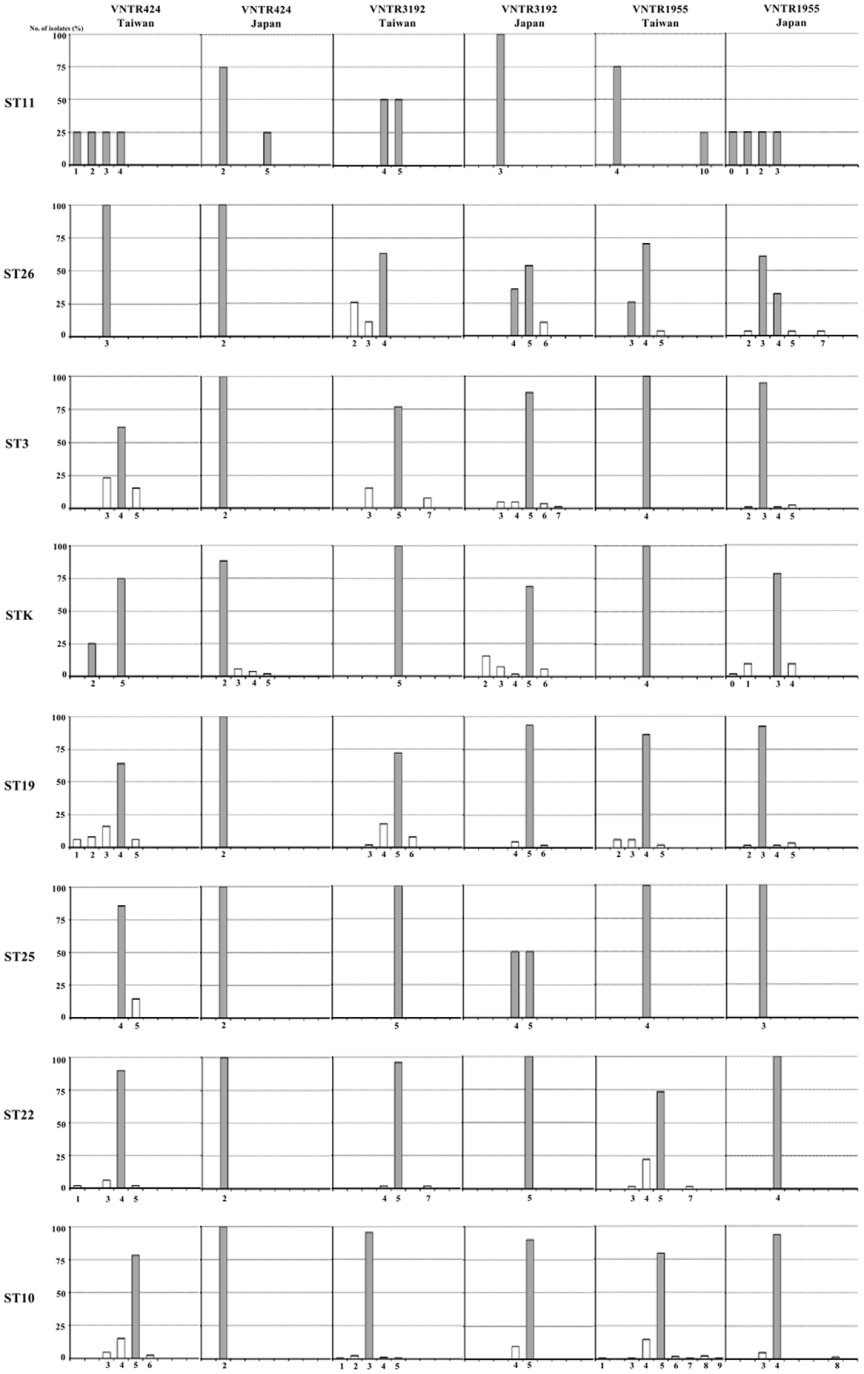
Allelic distribution of three VNTR loci in Taiwan and Japan. The repeat numbers of each VNTR locus are shown as gray and white bars for the numbers of isolates that comprised >25% and <25% of the proportion, respectively.

In an attempt to address the evolution of Beijing sublineages, the proportions of each Beijing sublineage were categorized according to the number of repeats of a particular VNTR locus. Three of the loci (VNTR424, VNTR3192 and VNTR1955) were phylogenetically informative (Fig. 1). Evolution of the ancestral or modern Beijing subfamilies, based on the repeat number of VNTRs, was also revealed (ancestral or modern Beijing strains were classified by determining the presence of an RD deletion as described in our previous study [16]). For example, VNTR3192 of Taiwan was characterized mainly by 4 repeats in ST11 and ST26 (ancestral subfamily), but showed almost exclusively 5 repeats in ST3, STK, ST19, ST25, ST22 (modern subfamily) (Fig. 1). VNTR1955 of Japan showed mainly 3 repeats in ST11, ST26, ST3, STK, ST19 and ST25 (ancestral subfamily), but 4 repeats in ST22 and ST10 (modern subfamily) (Fig. 1).

Furthermore, the proportions of 3 repeats or 4 and 5 repeats in VNTR424 of Taiwan demonstrated high specificity and sensitivity with respect to the classification of Beijing subfamilies (Fig. 1) (3 repeats distinguished the ancestral Beijing strains: sensitivity 28/31, 90%; specificity 22/307, 93%; 4 and 5 repeats distinguished the modern Beijing strains: sensitivity 272/307, 89%; specificity 1/31, 97%).

### Phylogenetic Analysis of Beijing Family Isolates from Taiwan, Japan, and South Korea

**Figure 2:**
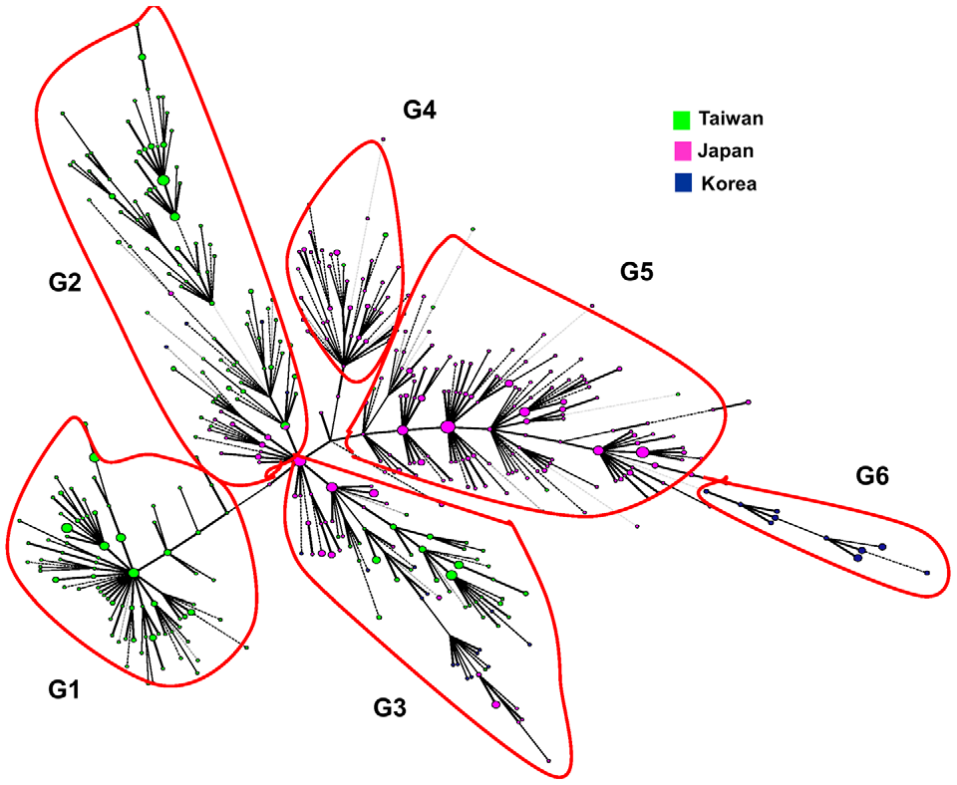
A minimum spanning tree based on 21–MIRU-VNTR genotyping of MTB isolates from Taiwan, Japan, and South Korea. The sizes of the branches correspond to the number of isolates with a particular genotype. Each country is assigned a different color. MTB isolates from Taiwan, Japan, and South Korea are colored green, pink, and blue, respectively.

Japan, South Korea and Taiwan are geographically close to mainland China. In addition to geographic nearness, Japan, South Korea, and Taiwan have had historical relationships with mainland China. Business and cultural exchanges among these countries began thousands of years ago. In World War II, Japan colonized Taiwan and Korea. Chinese characters are also used in these three countries. In view of these economic and cultural ties, we compared the MTB strains that have been studied in these three countries. Figure 2 shows the genotype distributions in Taiwan, Japan and South Korea, represented graphically by different colors. Interestingly, the isolates from Taiwan (shown in green) are mostly found in three clusters (G1–G3). We analyzed the allelic diversity and distribution pattern of VNTR loci of the six major phylogenetic sublineages (G1–G6). The G1 sublineage, composed of modern Beijing strains, is predominant in Taiwan. Sublineages G2 and G3, which also contain mainly modern Beijing strains, localize to Taiwan and Japan. Sublineages G4 and G5, composed of predominantly ancient Beijing lineages, are present mainly in Japan. Sublineage G6, also composed predominantly of ancient Beijing strains, is localized in South Korea.

## Discussion

The Beijing strain is one of the most prominent MTB lineages in East Asia and has spread to other countries worldwide [22], [23]. The reasons for the apparent global success of the Beijing strain are not understood, but could include a variety of host-related factors, such as human population movements [5], selective pressure due to increased worldwide BCG vaccine coverage [7], and ineffective treatment of drug-resistant strains, leading to increased transmission.

Associations of different MTB lineages with different geographic populations have been described [24]. Here we show that geographical clustering is also observed within the Beijing lineage (Fig. 2). The G1, G2 and G3 groups, which contain mainly modern Beijing strains, localize to Taiwan and Japan (Fig. 2). However, G4, G5 and G6, composed predominantly of ancient Beijing strains, are localized in Japan and South Korea (Fig. 2).

As shown in this study, the most frequent strains in Taiwan were ST10 (53.25%), followed by ST19 (14.79%) and ST22 (14.50%), similar to the frequencies in Thailand (ST10, 56.67%; ST22, 17.18%; and ST19, 17.18%) (Table 3). Taiwan is a relatively isolated island. As a similar ST pattern occurred in Taiwan and Thailand, it might be due to frequent travel and immigration between the two countries in the last decade.

The modern Beijing sublineages have been more predominant than the ancestral sublineages throughout other Asian countries, including South Korea, Vietnam, Malaysia and Mongolia [1]. However, Wada and Iwamoto demonstrated that the Beijing family strains in Japan belong mainly to the ancestral subfamily [25]. According to Wada et al. [18], the most frequent strain in Japan was ST19 (31.27%), followed by ST3 (23.66%) and STK (14.37%), which belong to the ancestral subfamily (Table 3). The proportion of ancestral sublineages was similar between Taiwan (26.04%) and Thailand (20.86%) (Table 3), and significantly higher in Japan (78.87%).

Some epidemiological studies have indicated that MTB genotype distribution is highly related to geography, human population and ethnicity [15], [24], [26]. The 12-loci MIRU pattern of one of the strains from our previous investigation was determined to be 223325173533, which is the most predominant pattern for the classical Beijing type (ST1) in Taiwan [14]. Interestingly, this strain was also found in other countries, including Russia, China, and Vietnam [27]. Sequence typing permitted us to further subdivide this major group of strains into ST3, ST10, ST19, ST22 and ST25 in our studies. We believe that these modern Beijing strains, with their high degree of transmissibility, are currently spreading throughout the world. It was previously reported that the BCG vaccination favors the positive selection of modern Beijing strains [28]. Our results support this finding. Moreover, an important study by Reed et al. [29] showed that 80% of the strains from modern Beijing sublineages, but not from ancient sublineages, synthesize relatively high quantities of phenolic glycolipid (PGL), which suppresses proinflammatory cytokines. These findings suggest that modern sublineages may be more pathogenic than ancient sublineages.

The predominance of ancient Beijing lineages in Japan may be because Japan is an island nation and human interaction with foreign countries was probably largely restricted until the 19^th^ century. In fact, TB was not reported as a prevalent infectious disease in Japan until the mid-20^th^ century. But the predominance of modern Beijing sublineages that has occurred recently in countries outside of Japan [30], [31] demonstrates the importance of uncovering the nature of the modern sublineages that has led to their worldwide prevalence and transmission.

The present study also gives the first overview of the evolution of Beijing sublineages in Taiwan based on analyzing the number of repeats of a particular VNTR locus. In our study, three evolutionarily informative loci (VNTR424, VNTR3192 and VNTR1955) were identified. We found that these loci had an increasing trend in the number of repeats (Fig. 1), whereas the remaining VNTRs showed an unvarying number of repeats (data not shown). According to the results, most of the VNTR loci exhibited small variations in the number of repeats (data not shown), which makes it difficult to construct a phylogenetic tree. Notably, the evolution of repeat number in VNTR1955 from Taiwan, Japan and Thailand [21] presented the same trend, which showed a constant repeat number in all STs (principally 4 repeats in Taiwan, 3 repeats in Japan and Thailand) but with increasing numbers in ST22 and ST10 (principally 5 repeats in Taiwan, 4 repeats in Japan and Thailand) (Fig. 1). We propose that the evolutionary history of Beijing isolates in these regions might involve the same events. We also demonstrated the evolutionary pathway of the Beijing strains based on repeat numbers of VNTRs. For example, VNTR424 showed 3 repeats in ST11 and ST26 (ancestral strains), which increased to 4–5 repeats in all other STs that belong to the modern subfamily. VNTR3192 also showed a similar trend (principally 4 repeats in the ancestral sublineages, 5 repeats in the modern sublineages).

Phylogenetic clues in the studied East Asian countries were also revealed (Fig. 2). The NTF and RD analyses in Taiwan indicated that ancient Beijing strains are prevalent among the aboriginals, and modern Beijing strains predominate among military veterans and the general population [15]. The major ethnic population associated with the G1 sublineage of Taiwan, which consists of mainly modern Beijing strains, is Han Chinese (classification by ethnic population in the sampled regions). Sublineage G2, also composed of mainly modern strains, is found predominantly among Han Chinese and military veterans. Taiwan was continuously occupied by the Japanese from 1895 until 1945. The five Taiwanese patients in the G3 sublineage (belonging to the ancient subfamily) were all aboriginals. Therefore, the MTB isolates of the G3 sublineage in Taiwan probably originated in Japan. The minimal spanning tree constructed in this study may constitute a good model with which to study MTB transmission.

In conclusion, this study compares the evolutionary pathway of MTB Beijing isolates from Taiwan against published profiles from three other Asian countries. The classification of MTB Beijing sublineages based on VNTRs was also described. This study also supports the clonal evolution of *M. tuberculosis* lineages in Taiwan and gives the first overview of the evolution of the Beijing family of MTB from neighboring Asian countries. Our study may be useful in epidemiological studies of global MTB Beijing isolates.

## Materials and Methods

### Bacterial Strains

A total of 338 Beijing strains was collected during 2003 to 2007 from four geographical regions of Taiwan (north, east, central and south) as described in our previous publication [16]. All 338 isolates were genotyped by spacer oligonucleotide typing (spoligotyping) and 24-locus MIRU-VNTR typing (Table S1). The *M. tuberculosis* strain H37Rv was used as the control.

### DNA Extraction

Mycobacterial chromosomal DNA was extracted by boiling a cultured cell suspension scraped from Lowenstein-Jensen slants in 200 µl distilled water at 85°C for 30 minutes. After centrifugation, the supernatants containing DNA were removed and stored at −20°C until further use.

### 24-locus MIRU-VNTR Typing

The 12 classical MIRU-VNTR loci (‘12-locus’), 3 exact tandem repeats (ETR A, B and C) and 9 additional loci (Mtub04, Mtub21, Mtub29, Mtub30, Mtub34, Mtub39, QUB11b, QUB26 and QUB4156) were selected and individually amplified in all 338 Beijing isolates as previously described by Supply et al. [32]. The resulting typing pattern from the 24 loci was used to create a 24-digit allelic profile for each isolate.

### SNP Typing

PCR procedures and extension primers were designed as described in our previous publication [16]. SNP typing at 10 loci was based on the genome sequences of the H37Rv strain [2], [17].

### Statistical Analysis

The Hunter-Gaston diversity index (HGDI) was calculated as described previously [33] and used for comparison of the discriminatory power of MIRU-VNTR typing of Beijing strains from each of the studied East Asian countries. According to this index, the loci were designated as highly (HGDI>0.6), moderately (HGDI 0.3–0.6) and poorly discriminatory (HGDI <0.3) as described in Sola et al. [19].

## Supporting Information

Table S1 24-MIRU-VNTR and SNP profiles of 338 Beijing isloates in this study.
(XLS)
